# Gram-negative bacteria and their lipopolysaccharides in Alzheimer’s disease: pathologic roles and therapeutic implications

**DOI:** 10.1186/s40035-021-00273-y

**Published:** 2021-12-07

**Authors:** Hyeon soo Kim, Sujin Kim, Soo Jung Shin, Yong Ho Park, Yunkwon Nam, Chae won Kim, Kang won Lee, Sung-Min Kim, In Duk Jung, Hyun Duk Yang, Yeong-Min Park, Minho Moon

**Affiliations:** 1 Department of Biochemistry, College of Medicine, Konyang University, Daejeon, 35365 Republic of Korea; 2 Research Institute for Dementia Science, Konyang University, Daejeon, 35365 Republic of Korea; 3 Dandi Bioscience Inc, 6th Floor of Real Company Building, 66, Achasan-ro, Sungdong-gu, Seoul, Republic of Korea; 4 Harvard Neurology Clinic, 294 Gwanggyojungang-ro, Suji-gu, Yongin, 16943 Republic of Korea; 5 Department of Immunology, School of Medicine, Konkuk University, 268, Chungwondaero, Chungju-si, Chungcheongbuk-do Republic of Korea

**Keywords:** Alzheimer’s disease, Gram-negative bacteria, Lipopolysaccharide, Exotoxin, Amyloid beta, Tau

## Abstract

Alzheimer’s disease (AD) is the most serious age-related neurodegenerative disease and causes destructive and irreversible cognitive decline. Failures in the development of therapeutics targeting amyloid-β (Aβ) and tau, principal proteins inducing pathology in AD, suggest a paradigm shift towards the development of new therapeutic targets. The gram-negative bacteria and lipopolysaccharides (LPS) are attractive new targets for AD treatment. Surprisingly, an altered distribution of gram-negative bacteria and their LPS has been reported in AD patients. Moreover, gram-negative bacteria and their LPS have been shown to affect a variety of AD-related pathologies, such as Aβ homeostasis, tau pathology, neuroinflammation, and neurodegeneration. Moreover, therapeutic approaches targeting gram-negative bacteria or gram-negative bacterial molecules have significantly alleviated AD-related pathology and cognitive dysfunction. Despite multiple evidence showing that the gram-negative bacteria and their LPS play a crucial role in AD pathogenesis, the pathogenic mechanisms of gram-negative bacteria and their LPS have not been clarified. Here, we summarize the roles and pathomechanisms of gram-negative bacteria and LPS in AD. Furthermore, we discuss the possibility of using gram-negative bacteria and gram-negative bacterial molecules as novel therapeutic targets and new pathological characteristics for AD.

**Supplementary Information:**

The online version contains supplementary material available at 10.1186/s40035-021-00273-y.

## Introduction

Alzheimer's disease (AD), one of the main causes of dementia, is a neurodegenerative disease causing cognitive decline and impairment of memory, language, and attention [1]. Previous studies have demonstrated that amyloid-β (Aβ) and tau are pathological hallmarks and primary causes of AD [2]. Medications targeting Aβ and tau have been developed to treat AD; however, they have not been effective in clinical trials. Therefore, changes in therapeutic targets are required, and upstream pathogenic contributors that affect Aβ and tau pathology are receiving increasing interest [3–5]. Many studies regarding the relationships between microbes and AD have indicated microorganisms as one of the new therapeutic targets for AD [6–8]. Namely, extensive changes in the microbiome occur in AD, and studies analyzing the intestinal microbiome in AD patients and animal models have provided interesting insights [9, 10].

Various sources of infection, such as fungi, viruses, and bacteria, are reportedly associated with AD [7]. The occurrence of microbiome dysbiosis or infection of several species of toxic bacteria may contribute to AD pathogenesis by triggering strong inflammatory responses or participating in Aβ production [11, 12]. Surprisingly, systemic inflammatory responses due to bacterial infection may also continue, causing neuronal cell death and Aβ/tau accumulation, contributing to the development and progression of AD. The brains of AD patients contain 5–10 times more bacteria than healthy brains; differences in the distribution and composition of the bacteria have also been studied [13]. In addition, it has been reported that the gram-negative bacteria can cross the blood–brain barrier (BBB) and contribute to neuroinflammation, Aβ accumulation, and tau hyperphosphorylation within the brain [14]. In particular, several gram-negative bacteria, such as *Proteobacteria* and *Chlamydophila pneumonia*, have been reported to provoke strong systemic inflammation and contribute to AD pathogenesis [15, 16]. Furthermore, the byproducts of gram-negative bacteria, such as lipopolysaccharides (LPS), capsular proteins, fimbrillins and flagellins, can also penetrate the brain and affect neuroinflammation, and Aβ and tau pathology [17].

LPS is a macromolecule mainly distributed in the outer membrane of gram-negative bacteria and acts as a powerful endotoxin [18]. The immune system responds with high sensitivity to LPS, high concentrations of which can cause sepsis and septic shock [19]. Furthermore, sepsis caused by LPS is a risk factor for cognitive impairment and AD development [20, 21]. Interestingly, LPS concentration in the plasma of patients with AD is 3–6 times higher than normal; similarly, LPS concentration in the blood of AD animal models is approximately 3 times higher than normal [18, 22, 23]. In AD, the LPS-induced pro-inflammatory immunomodulation is suggested to have a fatal effect on AD pathology [24]. Furthermore, a vicious cycle involving infectious species and their products in the induction of AD pathology has been continually proposed as an ‘infection hypothesis’ that potentially provides interesting insights into AD pathology [5, 17, 25]. These pieces of evidence suggest that LPS could not only be one of the upstream pathologic processes that either initiate or induce AD pathology, but also a promising therapeutic target for AD treatment.

Despite accumulating evidence on the importance of gram-negative bacteria and their LPS in AD pathology, their pathogenic mechanisms have not been clarified. Therefore, we attempted to summarize the pathogenic roles of gram-negative bacteria and their LPS in AD pathology and discuss their potential as effective therapeutic targets for AD (Additional file 1: Fig. S1).

## Pathomechanisms of gram-negative bacteria in AD

### Alteration of gram-negative bacteria in AD

With a growing interest in the importance of intestinal bacteria in AD pathology, an increasing number of studies have found links among gut microbiota, infectious microbes, and AD pathogenesis. Particularly, gram-negative bacteria are associated with the onset of AD. Various studies have demonstrated a positive association between gram-negative bacteria, such as *Helicobacter pylori *(*H. pylori*), *Porphyromonas gingivalis *(*P. gingivalis*), *Prevotella melaninogenica*, and *Campylobacter rectus*, and incidence of AD [26–30]. Moreover, some studies showed that the presence of gram-negative bacteria is directly associated with AD mortality [27, 30, 31]. Surprisingly, there are many studies pointing to the changes in gram-negative bacterial composition in animal models and patients with AD (Tables 1 and 2). In 8-month-old amyloid precursor protein/presenilin 1 (APP/PS1) mice, the proportion of *Bacteroidetes* at the phylum level increases, while *Allobaculum* and *Akkermansia* decrease at the genus level; concurrently, there is an increase in *Rikenellaceae* and S24-7 [32] (Table 1). In addition, another study demonstrated that *Helicobacteraceae* and *Desulfovibrionaceae* at the family level are significantly higher in APP/PS1 mice than in wild-type (WT) mice [33]. At the genus level, *Helicobacter* and *Odorivacter* are significantly abundant in APP/PS1 mice, while *Prevotella* abundance is remarkably higher in WT mice. In APP/PS1 mice, the proportion of *Bacteroidetes* tends to increase as AD progresses [34]. In the case of 5×FAD mice, it has been confirmed that the distribution of intestinal gram-negative bacteria starts to change at 9 weeks compared to that in WT mice. The proportion of *Bacteroidetes* in 5×FAD mice tends to decrease at 9-week-old and 18-week-old compared to 3-week-old in 5×FAD mice. These results indicated that the proportion of gram-negative bacteria changes with AD progression in 5×FAD mice [35]. Furthermore, it is known that the distribution of intestinal gram-negative bacteria in healthy individuals differs from that in AD patients, and the distribution of intestinal gram-negative bacteria changes as AD progresses. Some analyses of microorganisms in blood and feces showed that patients with AD have changed populations of gram-negative bacteria, such as *Spirochetes, Chlamydia, Proteobacteria, Firmicutes, Bacteroidetes,* and *Actinobacteria* at the phylum level (Table 2). In particular, it has been confirmed that the population of *Firmicutes* and *Bifidobacterium* decreases and the population of *Bacteroidetes* increases in the feces of patients with AD, compared with healthy controls [9]. Surprisingly, gram-negative bacteria found in the peripheral nervous system have also been observed in the central nervous system (CNS) in patients with AD [13, 36–39]. The gram-negative bacteria that show changes in the proportion and population within the brain and cerebrospinal fluid (CSF) of patients with AD are *Chlamydia, Proteobacteria, Bacteroidetes,* and *Spirochetes* [13, 15, 38, 40–49]. These results suggest that the gram-negative bacteria in the peripheral system can penetrate the BBB and infiltrate the brain.

**Table 1:** Species of gram-negative bacteria exhibiting alteration in animal models of Alzheimer’s disease

Source	Subject	Method	Gram-negative bacteria						References
			Phylum	Class	Order	Family	Genus	Species	
Feces	APP/PS1 mice	PCR	*Proteobacteria*			*Helicobacteraceae* *Desulfovibrionaceae*	*Helicobacter*		[33]
			*Bacteroidetes*	*Bacteroidia*	*Bacteroidales*	*Porphyromonoadaceae*	*Odoribacter*		
		PCR	*Bacteroidetes*						[50]
		PCR	*Bacteroidetes*						[32]
			*Bacteroidetes*	*Bacteroidia*	*Bacteroidales*	*Rikenellaceae*			
			*Bacteroidetes*	*Bacteroidia*	*Bacteroidales*	*S24-7*			
			*Verrucomicrobia*	*Verrucomicrobiae*	*Verrucomicrobiales*	*Akkermansiaceae*	*Akkermansia*		
		PCR	*Bacteroidetes*						[34]
	5×FAD mice	PCR	*Proteobacteria*	*δ-, γ-, ε-Proteobacteria*		*Helicobacteriaceae, Pseudomonadaceae*			[23]
			*Bacteroidetes*	*Bacteroidia*	*Bacteroidia*	*Prevotellaceae*			
		PCR	*Bacteroidetes*						[35]
		PCR	*Bacteroidetes*	*Bacteriodia*	*Bacteroidales*	*Muribaculaceae*			[10]
	5×FAD mice 3×Tg mice	PCR	*Bacteroidetes*						[51]
			*Proteobacteria*						

*PCR* Polymerase chain reaction

**Table 2:** Species of gram-negative bacteria exhibiting alteration in patients with Alzheimer’s disease

	Subject	Source	Method	Gram-negative bacteria						References
				Phylum	Class	Order	Family	Genus	Species	
Peripheral system	AD patients	Blood	ELISA	*Spirochetes*	*Spirochetes*	*Spirochetes*	*Spirochetaceae*	*Borrelia*	*Borrelia burgdorferi*	[52]
				*Chlamydia*	*Chlamydiae*	*Chlamydiales*	*Chlamydiaceae*	*Chlamydophila*	*Chlamydia pneumoniae*	
				*Proteobacteria*	*Epsilonproteobacteria*	*Campylobacterales*	*Helicobacteraceae*	*Helicobacter*	*Helicobacter pylori*	
			WB	*Spirochetes*	*Spirochetes*	*Spirochetes*	*Spirochetaceae*	*Borrelia*	*Borrelia burgdorferi*	[53]
			ELISA WB	*Spirochetes*	*Spirochetes*	*Spirochetes*	*Spirochetaceae*	*Borrelia*	*Borrelia burgdorferi*	[46]
		Feces	PCR	*Firmicutes*	*Negativicutes*	*Selenomonadales*	*Veillonellaceae*	*Dialister*		[9]
					*Negativicutes*	*Acidaminococcales*	*Acidaminococcaceae*	*Phascolarctobacterium*		
				*Bacteroidetes*	*Bacteroidia*	*Bacteroidales*	*Bacteroidaceae*	*Bacteroides*		
							*Rikenellaceae*	*Alistipes*		
				*Actinobacteria*	*Actinobacteria*	*Bifidobacteriales*	*Bifidobacteriaceae*	*Bifidobacterium*		
					*Actinobacteria*	*Coriobacteriales*	*Coriobacteriaceae*	*Adlercreutzia*		
				*Proteobacteria*	*Deltaproteobacteria*	*Desulfovibrionales*	*Desulfovibrionaceae*	*Bilophila*		
Central system	AD patients	Brain	PCR EM IEM	*Chlamydia*	*Chlamydiae*	*Chlamydiales*	*Chlamydiaceae*	*Chlamydophila*	*Chlamydia pneumoniae*	[40]
			PCR	*Proteobacteria*	*Gammaproteobacteria*	*Pseudomonadales*	*Methylobacteriaceae* *Moraxellaceae* *Bradyrhizobiaceae* *Sphingomonadaceae* *Comamonadaceae* *Xanthomonadaceae*			[13]
				*Bacteroidetes*						
			PCR	*Chlamydia*	*Chlamydiae*	*Chlamydiales*	*Chlamydiaceae*	*Chlamydophila*	*Chlamydia pneumoniae*	[41]
			PCR	*Spirochetes*	*Spirochaetes*	*Spirochaetes*	*Spirochaetaceae*	*Borrelia* *Treponemas*	*Borrelia burgdorferi* *T. pectinovorum* *T. amylovorum* *T. lecithinolyticum* *T. maltophilum,* *T. medium* *T. socranski*	[42]
			PCR	*Chlamydia*	*Chlamydiae*	*Chlamydiales*	*Chlamydiaceae*	*Chlamydophila*	*Chlamydia pneumoniae*	[43]
			EM AFM	*Spirochetes*						[44]
			PCR	*Chlamydia*	*Chlamydiae*	*Chlamydiales*	*Chlamydiaceae*	*Chlamydophila*	*Chlamydia pneumoniae*	[45]
			IF	*Bacteroidetes*	*Bacteroidia*	*Bacteroidales*	*Porphyromonadaceae*	*Porphyromonas*	*P. gingivalis*	[38]
				*Spirochetes*	*Spirochetes*	*Spirochete*	*Spirochetaceae*	*Treponema*	*T. denticola* *T. forsythia*	
			WB	*Spirochetes*	*Spirochetes*	*Spirochetes*	*Spirochetaceae*	*Borrelia*	*Borrelia burgdorferi*	[46]
			WB ICC PCR	*Proteobacteria*	*Gammaproteobacteria*	*Enterobacterales*	*Enterobacteriaceae*	*Escherichia coli*		[15]
		CSF	PCR	*Chlamydia*	*Chlamydiae*	*Chlamydiales*	*Chlamydiaceae*	*Chlamydophila*	*Chlamydia pneumoniae*	[47]
			ELISA	*Proteobacteria*	*Epsilonproteobacteria*	*Campylobacterales*	*Helicobacteraceae*	*Helicobacter*	*Helicobacter pylori*	[48]
			Serological analysis ELISA WB	*Spirochetes*	*Spirochetes*	*Spirochetes*	*Spirochetaceae*	*Borrelia*	*Borrelia burgdorferi*	[46]
			Histopathologic analysis	*Spirochetes*						[49]

*AD* Alzheimer’s disease, *AFM* atomic force microscopy, *CSF* cerebrospinal fluid, *ELISA* enzyme-linked immunosorbent assay, *EM* electron microscopy, *ICC* immunocytochemistry, *IEM* immunoelectron microscopy, *IF* immunofluorescence, *IHC* immunohistochemistry, *PCR* polymerase chain reaction, *WB* western blot

**Fig. 1:**
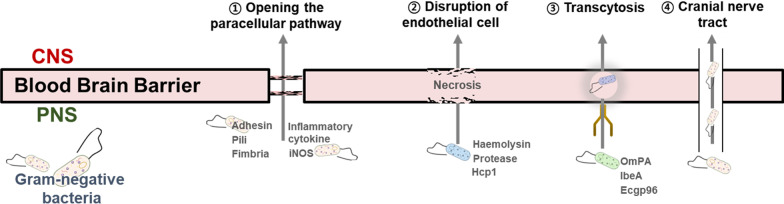
Mechanisms of gram-negative bacteria penetration to the central nervous system. ① The gram-negative bacteria-derived exotoxins provoke detachment of endothelial cells, and the gram-negative bacteria-induced inflammatory cytokines induce disruption of the tight junction at the blood-brain barrier (BBB). These impairments of BBB allow the gram-negative bacteria to pass through the brain in the paracellular pathway. ② The gram-negative bacteria-derived exotoxins directly influence endothelial necrosis. ③ The gram-negative bacteria are transported to the brain via vesicular transport of macromolecules, such as outer membrane protein A (OmPA), invasion of the brain endothelium protein A (IbeA), endothelial receptors beta-form of the heat-shock gp96 (Ecgp96), and contactin-associated protein 1 (CaspR1). ④ The cranial nerve can be a pathway for gram-negative bacteria to enter the brain without penetrating the BBB. CNS: Central nervous system; iNOS: Inducible nitric oxide synthase; PNS: peripheral nervous system

Interestingly, the gram-negative bacteria penetrate the BBB and affect the brain through four potential mechanisms (Fig. 1). First, gram-negative bacteria disrupt the intercellular junctions and induce endothelial cell detachment using their adhesin, pili, and fimbria, allowing them to penetrate the BBB through the paracellular pathway [54]. For instance, the pilus‐mediated signaling events promote alterations in tight junction organization. *Escherichia coli *(*E. coli*) binds to brain microvascular endothelial cell receptors to trigger the release of interleukin (IL)-6 and IL-8, and production of inducible nitric oxide synthase (iNOS), and at the meantime breaks down tight junctions between endothelial cells to aggravate BBB disruption [54]. Second, gram-negative bacteria can penetrate the BBB and enter the brain through necrosis of endothelial cells caused by exotoxins, such as hemolysin and protease. Injection of heme carrier protein 1, a component of the Type VI secretion system of *E. coli* K1, into the cytoplasm of human brain endothelial cells induces necrosis of endothelial cells [55]. Third, gram-negative bacteria internalize into the BBB through transcytosis via interactions between bacterial outer membrane proteins and endothelial cells [55, 56]. The *E. coli* K1 promotes bacterial transcytosis across the endothelium through outer membrane protein A (OmPA), invasion of the brain endothelium protein (IbeA), endothelial receptors beta-form of the heat-shock gp96 (Ecgp96), and contactin-associated protein 1. The gram-negative bacteria that enter the brain through transcytosis can survive and proliferate by reducing or escaping from the immune response. Finally, the gram-negative bacteria can enter the CNS through the cranial nerves. In particular, the trigeminal and olfactory nerves are suggested as major paths for oral bacteria to enter the brain [57], with several gram-negative oral bacteria capable of affecting the CNS through these neural tracts [58]. Studies using BALB/c mice and the AD model showed that oral bacteria migrate to the brain at a very high frequency through the cranial nerve [59, 60]. Furthermore, it has been reported that DNA of *P. gingivalis*, which is a gram-negative oral anaerobe involved in the pathogenesis of periodontitis, is characteristically detected in the brains and CSF of patients with AD [59]. In addition, *H. pylori,* a gram-negative bacterium, can enter the CNS through the oral–nasal–olfactory pathway or the gastrointestinal tract–brain neural pathway [61]. These studies support the hypothesis that the gram-negative bacteria can directly penetrate the brain. In this respect, the gram-negative bacteria penetrating the BBB can affect the onset or progression of AD.

### Possible roles of gram-negative bacteria in AD pathogenesis

#### Microbiota dysbiosis

Under healthy conditions, most intestinal microbiomes interact with the brain through several mechanisms, including neurotransmitter generation, and contribute to the maintenance of brain homeostasis [62]. Increasing evidence suggests that the gastrointestinal tract is the bridge between the microbiota and the CNS [63]. The link between the microbiome and brain disorders emerged from the impact of gastrointestinal microbes on the development of microbial byproducts in the brain [64]. The microbial-derived byproducts are active mediators of gut-brain communication and may be potential therapeutic targets for neurodevelopmental and neurodegenerative disorders. Particularly, bacterial byproducts and exotoxin molecules, such as capsular proteins, flagellin, short-chain fatty acids (SCFA), fimbrillin, peptidoglycan, proteases, gingipain, vacuolating cytotoxin A (VacA), and methylglyoxal (MG), may be considered pathogen-associated molecular patterns. Moreover, exotoxins interact with pattern recognition receptors, such as toll-like receptors (TLR) 2 and 4. Thus, bacteria imbalance and gut exotoxins induce neuroinflammatory reactions, such as microglial cell activation, affecting the function of neuronal cells [65]. The dysregulated microbiota-induced inflammation may also lead to the invasion of microbes or microbial byproducts into the brain, neuroinflammation, and production of Aβ and phosphorylated tau [66]. One study reported that alterations of the composition of gut microbiota in APP/PS1 mice are related to the increased Aβ levels in the brain and impairment of cognitive function. Moreover, another study found that the dysregulation of microbiota, intestinal epithelial barrier dysfunction, and vascular Aβ deposition occur in the intestine before the onset of cerebral Aβ deposition in Tg2576 mice [67]. These reports suggest that microbiota dysregulation is related to the development and progression of AD.

#### Aβ homeostasis

**Fig. 2:**
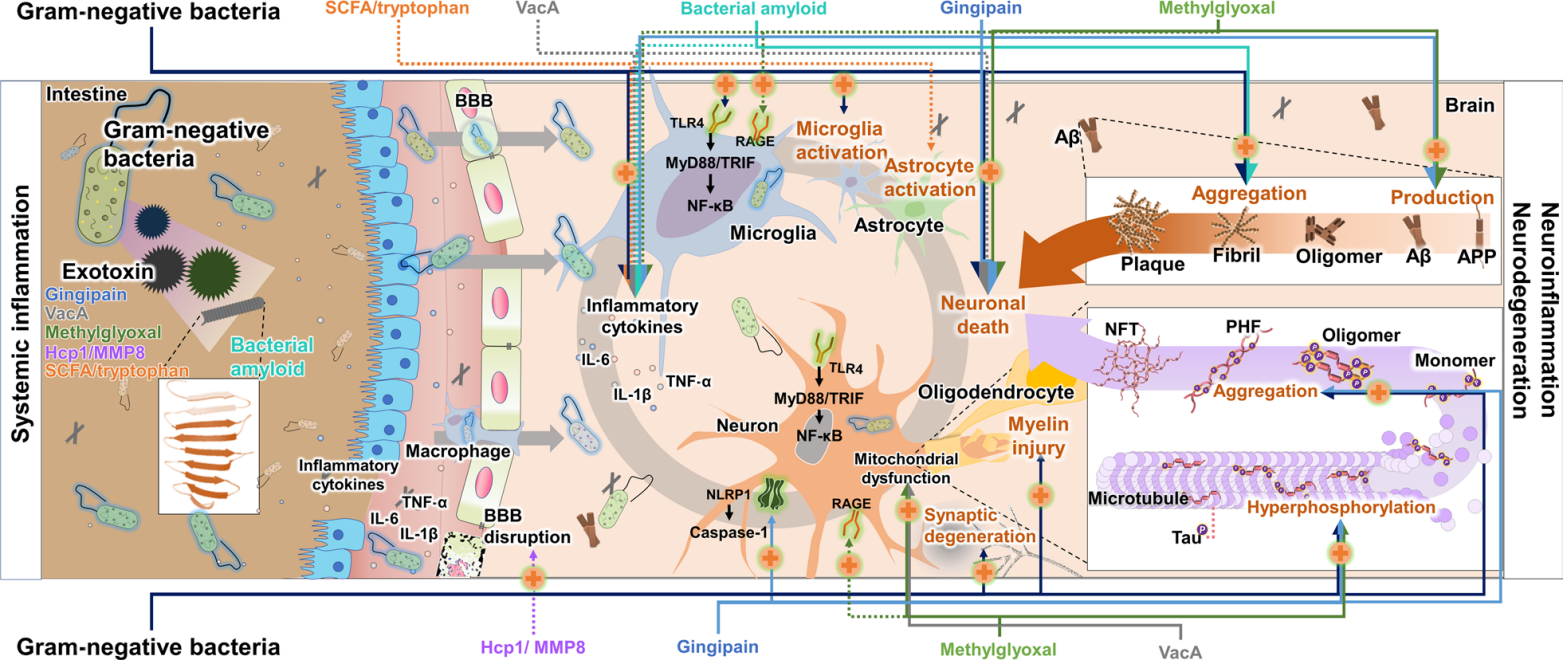
The pathological mechanisms underlying the effect of gram-negative bacteria in Alzheimer’s disease. The gram-negative bacteria produce a variety of exotoxins, such as gingipain, methylglyoxal (MG), bacterial amyloid, vacuolating cytotoxin (VacA), bacterial amino-acid, heme carrier protein (Hcp1), matrix metalloproteinase-8 (MMP8), phosphorylcholine, short-chain fatty acid (SCFA), and tryptophan. The gram-negative bacteria and exotoxins can penetrate the BBB and affect the AD-related pathology. Concerning Aβ aggregation, MG and gingipain are involved in the increase of Aβ production; bacterial amyloid and gram-negative bacteria can induce Aβ aggregation. Concerning hyperphosphorylated tau and neurofibrillary tangles, gingipain, MG, and gram-negative bacteria can provoke the hyperphosphorylation of tau; gingipain and gram-negative bacteria can also promote the aggregation of phosphorylated tau. Concerning neuroinflammation, the Aβ-induced activation of microglia and astrocytes contributes to a neuroinflammatory response, affecting neurodegeneration. The gram-negative bacteria and gingipain can increase the release of inflammatory cytokines. Concerning neurodegeneration, gingipain, MG, and gram-negative bacteria can induce neuronal death. The gram-negative bacteria provoke neuronal loss through the activation of the neuronal TLR4 signaling pathway. Hcp1: Heme carrier protein; IL-6: Interleukin 6; IL-1β: Interleukin 1β; IL-18: Interleukin 18; MMP8: Matrix metalloproteinase-8; NLRP1: Nod-like receptor protein 1; RAGE: Receptor for advanced glycation end products; SCFA: Short-chain fatty acid; TNF-α: Tumor necrosis factor α; VacA: Vacuolating cytotoxin A

The precipitate, which shows a β-folded sheet structure located vertical to the fibrous axis and is rich in aggregated insoluble lipoproteins, was designated as amyloid. Because of the hydrophobic nature of the aromatic amino acid peptides that compose the primary sequence of APP-derived amyloid, self-aggregation of amyloid monomers compiles over time into dimers, oligomers, and fibrils. Significant inflammatory reactions and neurodegeneration from amyloid accumulation appear in the brain, which affects neurodegenerative diseases, such as AD, Parkinson's disease, and prion disease [68]. Surprisingly, many studies have revealed the presence of bacteria-produced amyloids [68–70] (Fig. 2). The bacterial amyloids have a biophysical nature that is highly similar to human-derived amyloids, including the aggregate-forming ability [71, 72]. Although the amyloid produced by bacteria differs in its primary structure from the amyloid produced in the CNS, they have similarities in their tertiary structure [73]. Exposure to bacterial amyloid proteins in the gut could provoke systemic inflammation [74]. Moreover, bacterial amyloid affects disease progression by interacting with Aβ present in the AD brain (Table 3). The interaction of bacterial amyloid with neuronal amyloid, which is endogenously produced in the brain, promotes either their aggregation or cross-seeding in the AD brain [75, 76]. Furthermore, bacterial amyloid significantly promotes Aβ pathology in AD [69]. Curli is a well-known gram-negative bacterial amyloid. It is an integral part of the biofilm extracellular matrix produced by certain strains of *enterobacteria*, such as *E. coli* [77]. CsgA and CsgB are not only two major structural components of curli fibers, but are also essential components of biofilms [78]. In particular, the structure of the fibril produced by CsgA and CsgB of curli proteins is very similar to that of amyloid [79]. Interestingly, the fibril includes a β-sheet structure similar to Aβ and has been reported to form fibrillar aggregates [80]. The bacterial amyloid from curli may be a potential contributor to Aβ pathology in AD since several amyloid proteins interact with Aβ to induce co-aggregation or cross-seeding [37, 75]. In addition, FapC, a bacterial amyloid from *Pseudomonas*, is another strong contributor to Aβ pathology [81]. The FapC fibril is a powerful accelerator of Aβ fibrillization in AD [82]. In the co-culture of Aβ and FapC seeds, the rate of increase of the β-sheet ratio was more than three times above the Aβ-only culture [82]. Furthermore, FapC has been reported to promote Aβ-associated pathology several times faster in the AD zebrafish model [82]. Surprisingly, the bacterial amyloid and Aβ can bind to the same receptor due to their structural similarity. For example, similar to Aβ, the bacterial amyloid from curli can bind to the TLR2-TLR1-CD14 (cluster of differentiation 14) complex, which facilitates the nuclear factor kappa-light-chain-enhancer of activated B cells (NF-κB) pathway [83]. Moreover, MG, an intermediate metabolite of glucose metabolism in cells, is widely secreted by gram-negative bacteria such as *E. coli,* and distributed at a high rate in the body [84]. The MG acts as a ligand for the receptor for advanced glycation end products (RAGE), resulting in increased beta-site amyloid precursor protein cleaving enzyme 1 (BACE-1) expression and Aβ levels [85, 86]. In addition, it has been shown that *P. gingivalis* increases Aβ production by increasing the gene expression of APP and BACE1 and decreasing the gene expression of disintegrin and metalloproteinase domain-containing protein 10 (ADAM10) [59, 87]. Moreover, gingipain, a family of proteases secreted by *P. gingivalis*, can affect to activate γ-secretase through cleavage of caspase-3 [87]. In addition, three proteinase genes that contribute to the virulence of *P. gingivalis*, *RgpA*, *RgpB*, and *Kgp*, have been associated with Aβ production. In particular, *RgpB* has been reported to induce massive generation of Aβ by activating the metalloproteinase meprin β, which is an alternative BACE1 cleavage of APP [88]. Furthermore, the load of *KgpB* is particularly increased in the AD brain compared to the healthy brain, and RgpB has been reported to co-localize with Aβ [59]. Collectively, these findings suggest that bacterial molecules from gram-negative bacteria could induce the production and aggregation of Aβ, affecting the onset and progression of AD.

**Table 3:** Gram-negative bacteria-derived products affect Alzheimer’s disease and other diseases

	Gram-negative bacteria-derived products	AD-related pathology					References
		Aβ	Tau	Neuroinflammation	Cell death	BBB disruption	
In Alzheimer’s disease	Gingipain	Production ↑	Hyperphosphorylation ↑ Aggregation ↑	Proinflammatory cytokines ↑	Pyroptosis ↑ Caspase-1 ↑		[59]
	Methylglyoxal (MG)	Production ↑	Hyperphosphorylation ↑		Oxidative stress↑ Apoptosis↑		[64, 85, 89]
	Hcp1					Endothelial cell disruption ↑	[55]
	Bacterial amyloid	Aggregation ↑					[82]
In other diseases	VacA			Proinflammatory cytokines ↑	Cell vacuolation ↑		[90, 91]
	Bacterial amino-acid			Systemic inflammation ↑			[10]
	Hcp1					Endothelial cell disruption ↑	[55]
	MMP8					Junctional protein degradation ↑	[92]
	Phosphorylcholine			Proinflammatory cytokine ↑			[93]
	Short chain fatty acid			Proinflammatory cytokine ↑ Gliosis ↑		Endothelial cell disruption ↑	[94–96]
	Tryptophan			Proinflammatory cytokine ↑ Gliosis ↑		Endothelial cell disruption ↑	[97]

*AD* Alzheimer’s disease, *Aβ* Amyloid-β, *BBB* blood–brain barrier, *Hcp1* Heme carrier protein 1, *MMP8* matrix meralloproteinase-8, *ROS* reactive oxygen species, *VacA* vacuolating cytotoxin

#### Tau pathology

Hyperphosphorylated tau aggregation is the main pathological hallmark of AD [98]. Neurofibrillary tangles (NFTs) consist of hyperphosphorylated and aggregated microtubule-associated protein tau [99], while the intermediate form of tau causes cytotoxicity and cognitive impairment [100]. Tau hyperphosphorylation is regulated by various kinases that are affected by numerous factors, including gram-negative bacteria [101, 102] (Fig. 2). *Helicobacter pylori* induces tau hyperphosphorylation by activating the glycogen synthase kinase-3β (GSK-3β) [102]. Moreover, it has been revealed that *P. gingivalis* can increase tau hyperphosphorylation at, e.g., Thr231 and Ser396 residues, in human iPSC-differentiated neuronal cells and C57BL/6 mice [87, 103]. Particularly, *P. gingivalis* infection activates GSK-3β [104]. Furthermore, gingipain can contribute to tau hyperphosphorylation by regulating the protein kinase B (Akt)/GSK-3β activity by cleaving procaspase-3 to caspase-3 [59, 105]. Gingipain is involved in tau fragmentation and generation of paired helical filament through tau proteolysis; the tau fragments can induce tau aggregation and phosphorylation [106–108] (Table 3). Interestingly, Kgp has been reported as a trigger or accelerator of tau pathology [59]. The hexapeptide motif-containing tau peptide generated by Kgp can be easily hyperphosphorylated and contribute to the formation of paired helical filaments and NFT. Similarly, MG has been reported to induce tau hyperphosphorylation through the GSK-3β activity [89]. Furthermore, DNA derived from several species of gram-negative bacteria, such as *E. coli* and *P. gingivalis*, has been reported to promote tau pathology [109]. In particular, the gram-negative bacterial DNA—frequently reported in patients with AD—strongly induces tau misfolding and aggregation [109]. Therefore, gram-negative bacteria could initiate or exacerbate tau pathology by inducing tau hyperphosphorylation and aggregation in AD.

#### Neuroinflammation

Neuroinflammation is a pathological hallmark induced by abnormally aggregated Aβ peptides in AD [110, 111]. Moreover, microglia activated by Aβ can accelerate neurodegeneration in the brain during AD [111]. Gram-negative bacteria are known triggering factors for inflammatory responses [112]. A study confirmed that the effect of microbiota on microglial maturation in germ-free mice could be regulated by SCFA, a byproduct of bacterial metabolism [94]. Furthermore, the microbial metabolites of tryptophan can modulate astrocyte activity [97]. These results show that the bacterial-derived byproducts, such as VacA, SCFA, phosphorylcholine, and tryptophan [90, 94–97], are involved in neuroinflammation by modulating microglia and astrocyte activity. Although this evidence demonstrates the pivotal role of byproducts from gram-negative bacteria in neuroinflammation, only a few studies have investigated their effects in AD. A recent study showed that the *P. gingivalis* oral infection causes strong microglial activation in the brains of apolipotein E (ApoE)^−/−^ mice [60]. In particular, gingipain from *P. gingivalis* can lead to the release of neuroinflammatory cytokines in an AD brain [59] (Fig. 2). In addition, respiratory infection of *Bordetella pertussis* in APP/PS1 mice increases brain infiltration of T cells and activation of microglia and macrophages [113]. *Helicobacter pylori* infection stimulates the secretion of pro-inflammatory cytokines, such as tumor necrosis factor-α (TNF-α) and IL-6, causing inflammation-related neurodegeneration [61]. Moreover, it has been reported that monocytes infected with *Chlamydia pneumoniae *(*C. pneumoniae*) may contribute to late-onset AD by inducing secretion of pro-inflammatory cytokines and chemokines from microglia and astrocytes [114]. Furthermore, the C. *pneumoniae-*infected microglia show increased levels of TNF-α [115], a critical neuroinflammatory factor in AD. These findings imply that the gram-negative bacteria introduced into the CNS can aggravate AD pathology through strong neuroinflammation.

#### Neuronal cell death

Neuronal loss is a prominent pathological feature of AD [3]. Surprisingly, gram-negative bacteria, *H. pylori*, can induce neuronal cell death by secreting VacA [91]. Moreover, MG from gram-negative bacteria—a cell death-related toxin—has been reported to induce oxidative stress and apoptosis through activation of nicotinamide adenine dinucleotide phosphate oxidase and production of reactive oxygen species [64]. In AD, MG is a potential key factor that triggers neuronal death through the decrease of mitochondrial membrane potential, down-regulation of Bcl-2, and up-regulation of pro-apoptotic proteins, such as caspase-3 and Bax [116, 117]. These mitochondrial dysfunctions caused by MG can accelerate AD neurodegeneration. TLR4 activates the nod-like receptor protein 3 by recognizing gram-negative bacteria; it is a key receptor for the onset of neurodegenerative diseases, including AD [118]. Therefore, activation of TLR4 signaling by gram-negative bacteria can lead to neuronal cell death [119]. Importantly, gram-negative bacteria can trigger neuronal cell death through not only their byproducts but also the bacteria themselves (Fig. 2). Taken together, both gram-negative bacteria and their byproducts could play a key role in neuronal cell death and neurodegeneration in AD.

## Specific roles of LPS from gram-negative bacteria in AD

### Peripheral and central localization of LPS in AD

Most LPS is produced by intestinal gram-negative bacteria; subsequently, it can escape the intestine and enter the circulatory system [120]. The high concentration of LPS can induce TLR4-dependent CD14 upregulation in enterocytes, thereby damaging the intestinal epithelial barrier and increasing the gut permeability. Surprisingly, one study reported that the LPS level in the plasma is increased 3–6 times in AD patients (61 ± 42 pg/ml) compared to that in healthy controls (21 ± 6 pg/ml) [22]. Such changes could be associated with an increased permeability induced by LPS in the intestine [121, 122]. This suggests that, as AD increases, the intestinal epithelial barrier may have an increased permeability to intestinal LPS, which leads to the spread of LPS throughout the body.

**Fig. 3:**
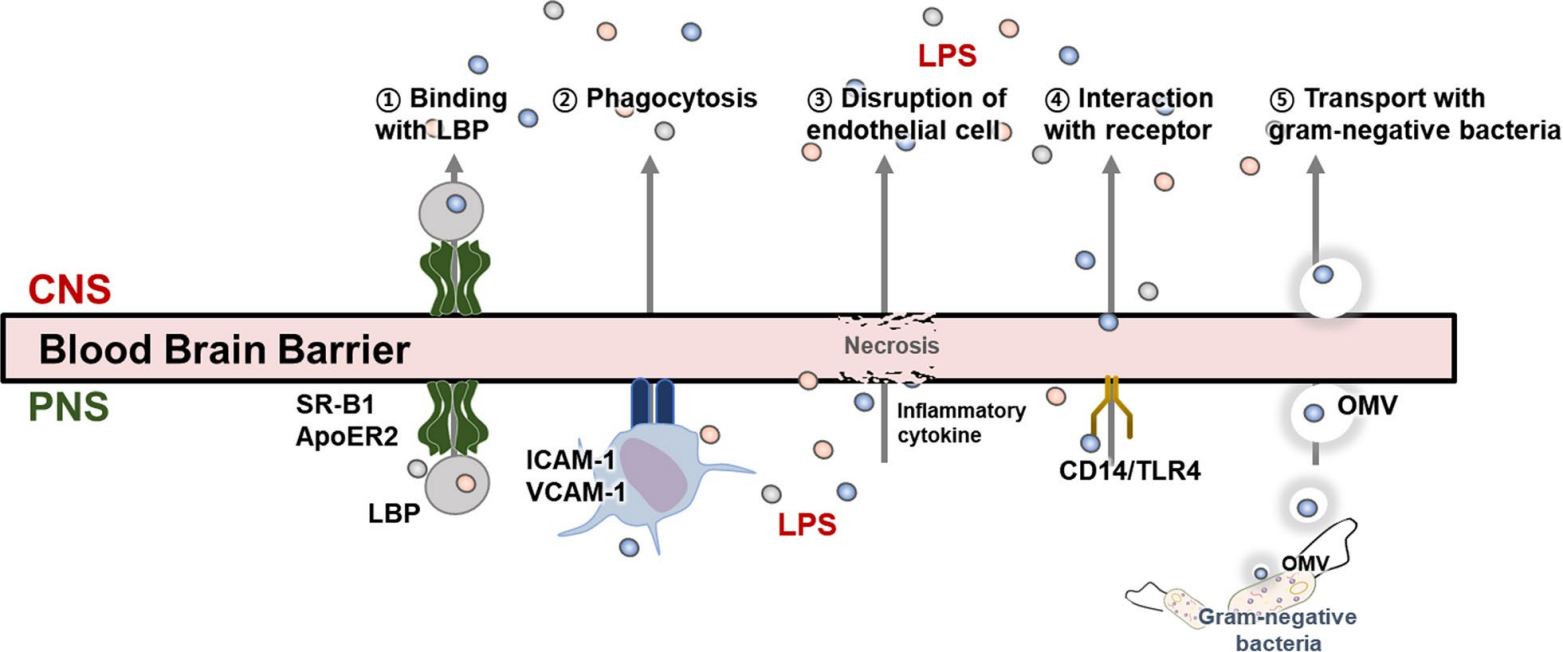
Mechanisms of lipopolysaccharide (LPS) penetration to the central nervous system. LPS produced in the peripheral system penetrates the BBB and enters the brain. ① LBP is a soluble acute-phase protein that binds to bacterial LPS to elicit immune responses. LBP facilitates LPS penetration of the BBB through various receptors, such as Scavenger reception class B type 1 (SR-B1) and apolipoprotein E receptor 2 (ApoER2). ② LPS is transported to BBB by peripheral immune cells. ③ LPS enters the brain via damaged BBB caused by high concentrations of LPS and LPS-induced pro-inflammatory cytokines. ④ LPS is directly recognized by the cell surface pattern recognition receptor CD14/TLR 14 complex, resulting in penetration to the BBB. ⑤ LPS is transported into the brain through gram-negative bacteria transporters, such as OMV. CD14: Cluster of differentiation 14; CNS: Central nervous system; LBP: Lipopolysaccharide-binding protein; OMV: Outer membrane vesicle; PNS: Peripheral nervous system; TLR4: Toll-like receptor 4; VCAM1: Vascular cell adhesion molecules-1

Surprisingly, several previous studies have shown an extensive and characteristic distribution of LPS in the AD brain (Table 4). LPS localization has been reported in various regions, such as the lateral ventricle of the parietal lobe and neocortex of the temporal lobe of the AD brain, suggesting that it can be widely distributed in AD brains [38, 123–125]. The mechanisms by which LPS crosses the BBB have not yet been clearly elucidated; however, several mechanisms have been proposed. First, LPS binds to the lipopolysaccharide-binding proteins (LBP) and can pass the BBB using receptors distributed within the BBB, such as scavenger receptor class B type I, apolipoprotein A-I and ApoE, and apolipoprotein E receptor 2 (ApoER2) [126] (Fig. 3). Second, LPS can be transported through phagocytosis by peripheral immune cells. In particular, LPS stimulation increases both the secretion of peripheral and central inflammatory cytokines and the expression of adhesion molecules in BBB endothelial cells, such as p-selectin, intercellular adhesion molecules-1, and vascular cell adhesion molecules-1, which potentially increases immune cell entry into the BBB [127–130]. Third, it has been suggested that high LPS doses induce a pro-inflammatory response, destroying the BBB and allowing LPS to enter the CNS [131]. For instance, either LPS or LPS-induced TNF-α can degrade glycocalyx in the endothelial cells of the BBB, thereby increasing the BBB permeability [132]. Fourth, LPS entry into the BBB could occur without mediation, by binding to the CD14/TLR4 complex on BBB endothelial cells [126]. Fifth, another interesting possibility is that the LPS molecules not only pass directly through the BBB [133] but also enter the brain through gram-negative bacteria [134], which are capable of transferring both exotoxins and endotoxins to the host cells through outer membrane vesicles (OMVs) [135]. An OMV is a bacterial transporter capable of entering various cell types, such as gut and BBB endothelial cells. Therefore, LPS can be introduced into neuronal cells through the OMV-containing LPS, derived from gram-negative bacteria. It is well established that the OMV-delivered LPS induces a stronger physiological response than pure LPS [135]. Therefore, LPS transmitted through gram-negative bacteria in the brain, can be more harmful to neuronal cells. Moreover, it is possible that LPS can accumulate in neuronal cells [124]. Taken together, LPS could sufficiently contribute to AD pathology through various BBB penetration mechanisms.

**Table 4:** Localization and change of lipopolysaccharides in Alzheimer’s disease

Source	Subject	Method	Main findings	References
Brain	AD patients	Immunoblot	LPS was detected in the area adjacent to the lateral ventricle of the parietal lobe of AD brain	[38]
	AD patients	WB IHC	LPS was detected in temporal lobe neocortex perinuclear region of AD brain LPS was co-localized with Aβ plaque	[125]
	AD patients	IF WB	LPS was detected in superior temporal gyrus gray matter, frontal lobe white matter, and periventricular white matter of AD brain LPS was localized with Aβ plaque, neurons, microglia, and oligodendrocytes	[15]
	AD patients	IHC	LPS was detected in superior temporal lobe neocortex of AD brain LPS was localized in neurons	[124]
	AD patients	WB	LPS was detected in temporal lobe neocortex and hippocampus of AD brain	[123]
	5×FAD mice	IF	LPS was detected in pyramidal and stratum oriens regions of hippocampus of AD brain LPS was co-localized with LPS-phagocytic cell	[23]
Blood	AD patients	LAL assay	LPS levels in AD patients were 3- to 6-fold compared with that in control	[22]
	5×FAD mice	ELISA	LPS levels in AD mice were 4-fold compared with that in control	[23]
	5×FAD mice	LAL assay	LPS levels in AD mice were 4-fold compared with that in control	[136]
Feces	5×FAD mice	LAL assay	LPS levels in AD mice were 3- to 4-fold compared with that in control	[23, 136]

*AD* Alzheimer’s disease, *ELISA* Enzyme-linked immunosorbent assay, *IF* immunofluorescence, *IHC* immunohistochemistry, *LAL assay* limulus amebocyte lysate assay, *LPS* lipopolysaccharides, *WB* western blot

### Pathogenic contribution of LPS to AD-related pathology

#### Aβ homeostasis

**Fig. 4:**
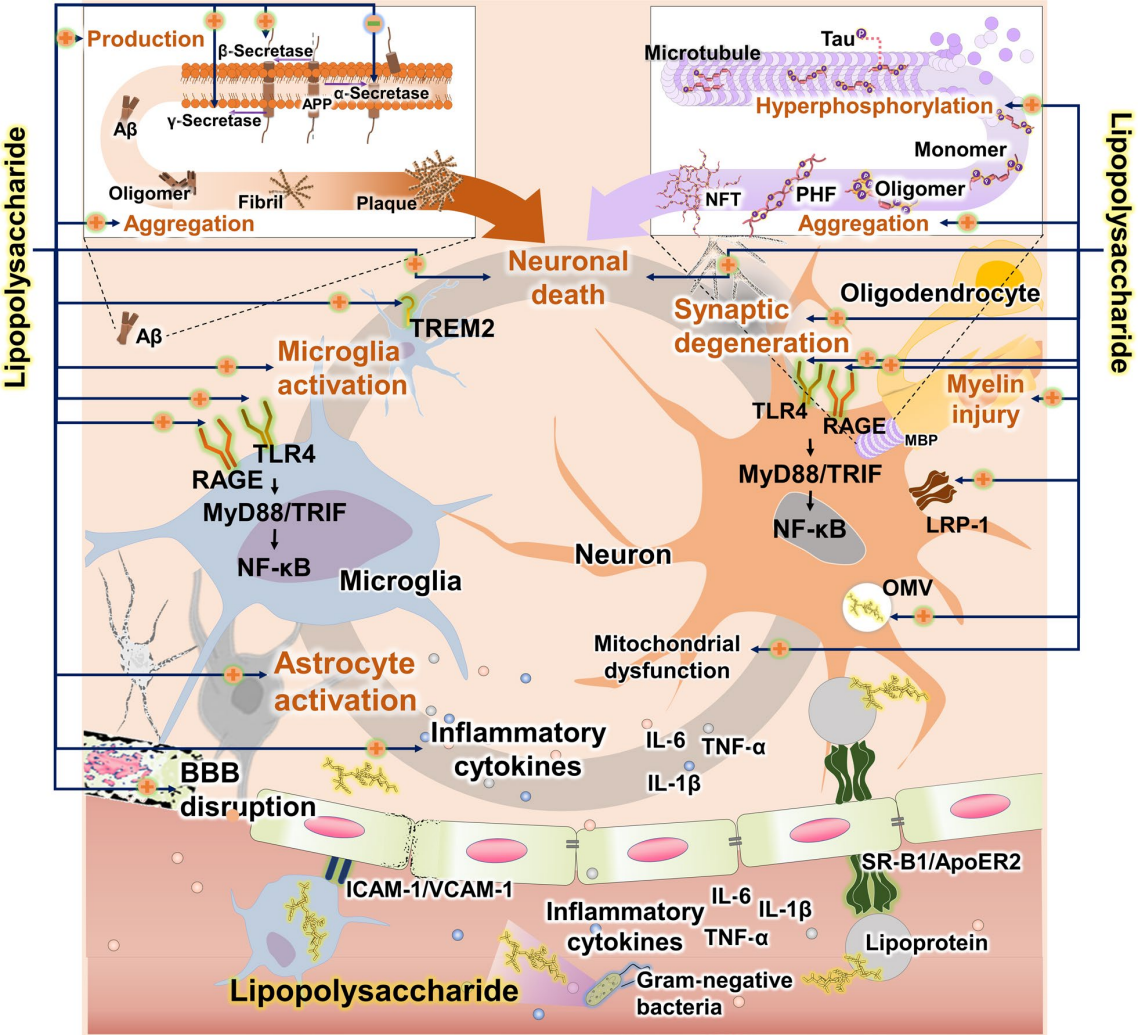
Pathogenic mechanisms of lipopolysaccharides (LPS) in Alzheimer’s disease. LPS is a characteristic component in the cell wall of gram-negative bacteria and plays a key role in triggering inflammatory response and initiating and promoting AD pathology. LPS promotes the production of Aβ through the increase of β- and γ-secretases and decrease of α-secretase, and stimulates the accumulation of Aβ. LPS induces the impairment of low-density lipoprotein receptor-related protein-1 (LRP-1), which plays a pivotal role in Aβ clearance. LPS is involved in tau phosphorylation, and accelerates the aggregation of phosphorylated tau. LPS activates the microglial TLR4, RAGE, and TREM2 receptors, inducing release of pro-inflammatory cytokines. LPS activation of the TLR4 signaling pathway and LPS entry in the brain through OMV can induce neuronal cell death. ApoER2: apolipoprotein E receptor 2; BBB: blood–brain barrier; IL-6: interleukin 6; IL-1β: interleukin 1 β; LRP-1: low-density lipoprotein receptor-related protein 1; MBP: myelin basic protein; MMP8: matrix metalloproteinase-8; MyD88: myeloid differentiation primary response 88; NF-κβ: nuclear factor kappa β; NFT: neurofibrillary tangles; NLRP1: Nod-like receptor protein 1; OMV: outer membrane vesicle; PHF: paired helical filament; RAGE: receptor for advanced glycation end products; SCFA: short-chain fatty acid; SR-B1: scavenger reception class B type 1; TLR4: Toll-like receptor 4; TNF-α: tumor necrosis factor α; TREM2: triggering receptor expressed on myeloid cells 2; TRIF: Toll/interleukin-1 receptor-domain-containing adapter-inducing interferon-β; VacA: vacuolating cytotoxin A; VCAM1: vascular cell adhesion molecules-1

Aβ accumulation is significantly reduced in sterile APP mice, but consistently increased in LPS-treated APP mice [32, 137], highlighting the role of LPS, apart from established AD pathogenic factors, and presenting LPS as a potential risk factor, equally strong as AD’s genetic components. LPS modulates Aβ production by significantly increasing the activity of APP-cleaving enzymes, such as BACE-1 and γ-secretase, while decreasing α-secretase activity [138] (Fig. 4). Moreover, LPS increases mRNA expression of APP and contributes to the production of Aβ in the hippocampus through the cathepsin B-related mechanism [139]. In LPS-injected rodents, BACE1 immunoreactivity and Aβ accumulation were found in the ipsilateral cerebral cortex and hippocampal formations [140]. Moreover, LPS increases Aβ oligomers by promoting Aβ aggregation [18, 141]. Furthermore, the LPS-induced systemic inflammation could provoke Aβ clearance impairment via (1) down-regulated expression of low-density lipoprotein receptor-related protein 1 (LRP-1); (2) inhibition of Aβ entry into the blood vessels in the brain; and (3) dysfunction of p-glycoprotein [142]. These studies demonstrate that the LPS-induced Aβ burden and Aβ plaques could play key roles in Aβ-related AD pathology.

#### Tau pathology

LPS is potentially instrumental in exacerbation of tau pathology (Fig. 4). First, LPS induces tau phosphorylation through not only hypoactivation of tau phosphatase but also activation of cyclin-dependent kinase 5 (CDK-5) and GSK-3β [143, 144]. LPS may stimulate the activation of GSK-3β through regulating the phosphoinositide-3-kinase (PI3K)/Akt pathway, increasing the production of phosphorylated tau [104, 145]. In particular, LPS can increase phosphorylation of tau in the hippocampus [144, 146, 147]. Second, LPS induces tau aggregation in the AD brain [143]. Several studies have reported that LPS contributes to the tau aggregation through activation of CDK-5 and GSK-3β in the 3xTg model [143, 144]. Accumulating evidence suggests that LPS accelerates tau pathology by acting as an exogenous regulator of kinases such as mitogen-activated protein kinase (MAPK), GSK-3β, c-Jun N-terminal kinases, and p38 [143, 148]. These results suggest that LPS may affect tau pathology by inducing hyperphosphorylation and aggregation of tau. Moreover, LPS could play a key role in tau hyperphosphorylation and aggregation, which is considered a major factor and therapeutic target for AD pathology.

#### Neuroinflammation

Accumulating evidence suggests that LPS contributes to AD pathology through glial activation modulation (Fig. 4). It has been reported that LPS administration increases microglial density in the brain [141]. In particular, LPS is an agonist of glial TLR4, activating the myeloid differentiation primary response 88/toll/interleukin-1 receptor-domain-containing adapter-inducing interferon-β (MyD88/TRIF) pathway and promoting pro-inflammatory responses by activating NF-κB [149–151]. In contrast, some studies have suggested that LPS promotes the anti-inflammatory response [152, 153]. It has been proposed that continuous mild LPS exposure reduces inflammatory responses in the brain by suppressing pro-inflammatory mediators and boosting anti-inflammatory mediators [152]. In addition, several studies have revealed that low-dose LPS can induce anti-inflammatory responses in AD animal models [154–156]. The possible underlying mechanism is that the low-dose LPS exposure reduces inflammation via upregulating the expression of MyD88-dependent signaling pathway inhibitors like IRAK-M, Ship, and Tollip [157]. Unfortunately, the precise mechanism by which mild LPS exposure provokes the anti-inflammatory response is not known. Although the role of LPS-exposed microglia in the AD brain is not clear, accumulating evidence suggests that LPS may exacerbate AD pathology through interactions with several receptors related to neuroinflammation [158, 159]. Notably, LPS significantly increases the expression level of RAGE [160], a receptor critically involved in AD pathology such as Aβ production and clearance, tau pathology, and synaptic degeneration [161]. In particular, the microglial RAGE-dependent signaling pathway plays a causative role in neuroinflammation, Aβ deposition, and cognitive impairment in AD [162]. Interestingly, it has been reported that stimulation of RAGE by LPS increases endothelial permeability and activates NF-κB [163]. The glial NF-κB activation by LPS leads to secretion of pro-inflammatory cytokines. In addition, recent studies have suggested that LPS can induce leukocyte infiltration into the brain and microglial activation by increasing the generation of reactive oxygen species through NADPH oxidase 2 (NOX2) activation [164]. The widespread high-level LPS in the AD brain alone can cause pathological and excessive neuroinflammatory reactions. Interestingly, LPS is also a ligand for triggering receptors expressed on myeloid cells 2 (TREM2), a receptor that regulates microglial phenotype switching [165]. Stimulation of TREM2 by LPS can convert the microglial phenotype from an anti-inflammatory phenotype to a pro-inflammatory phenotype [166]. Particularly, not only Aβ-induced neuroinflammation but also LPS-induced neuroinflammation could facilitate neurodegeneration and cognitive impairment [167]. These results suggest that the LPS-induced neuroinflammation could act as a direct and fatal factor in AD pathology and cognitive dysfunction.

#### Neurodegeneration

LPS induces synaptic loss within the CNS [168] and reduces synaptic plasticity in the brain [169]. Moreover, LPS affects the inhibitory and excitatory synapses of adult-born hippocampal neurons, induces neuronal and synaptic loss, and reduces cognitive function [170, 171], supporting the hypothesis that LPS in AD is involved in neurodegeneration (Fig. 4). In addition, LPS can inhibit neuronal function via damage to myelin in AD [172]. It has been reported that LPS causes damage to oligodendrocytes and increases myelin basic protein degradation in the AD brain. The first mechanism for LPS-induced neurodegeneration is that LPS induces the activated p38α MAPK signaling pathway in microglia and increases TNF-α secretion [173, 174]. In addition, LPS is one of the potent factors capable of activating NOX2 in the CNS [164, 175]. The NOX2 activation in glia and neurons can induce neuronal cell death through massive oxidative stress, which has been suggested as a contributor to several neurodegenerative diseases, including AD [176, 177]. The LPS-induced neuroinflammation such as NOX2 activation can be a possible additional contributor to neurodegeneration in AD pathology. Second, LPS facilitates neurodegeneration through mitochondrial dysfunction [178]; namely, LPS may affect mitochondrial fusion genes, such as mitofusin (Mfn)1, Mfn2, and OPA1, which are important in neurodegenerative diseases, including AD. In addition, LPS leads to neurodegeneration by inducing oxidative stress and triggering the mitochondrial apoptotic pathway [179]. Third, LPS can directly induce neuronal cell death through neuronal TLR4, which is a major receptor that plays a key role in the activation of the inflammatory response on AD. LPS not only increases TLR4 expression but also acts as a ligand for neuronal TLR4, inducing the transcription of caspase-11 and promoting the activation of the inflammasome [180, 181]. Since TLR4 expression is also increased by aging and Aβ, the interaction between LPS and TLR4 may be more fatal to AD [182]. NF-κB, a well-known downstream molecule of the TLR4/Myd88/TRIF signaling pathway, is also known to be important for neuronal survival and acts either as a pro-apoptotic or anti-apoptotic factor [183]. Consequently, LPS can cause neuronal death by directly acting on neurons, such as acting on neuronal receptors followed by influx into neuronal cells through OMV [181]. Taken together, these studies demonstrate that LPS can induce neurodegeneration and affect the initiation and progression of AD.

## Gram-negative bacteria and their LPS as therapeutic targets in AD

The paradigm of AD treatment research is transforming from identifying a single target towards a multi-target therapy for various pathogenic factors. Interestingly, several therapeutic approaches targeting LPS-releasing gram-negative bacteria and microbiota have been proposed.

### Antibiotics for AD treatment

Consistent with the influence of gram-negative bacteria on AD pathology, antibiotics have been demonstrated to reduce pathological changes in AD animal models and improve symptoms in AD patients (Table 5). Accumulating evidence on antibiotic therapy for AD suggests that the decrease of gram-negative bacteria involved in AD-related pathology by antibiotics is beneficial in the treatment of AD. However, some studies have suggested potential risk of side effects associated with their long-term use [184]. One of the largest risk factors is the antibiotic-induced microbiome imbalance [185]. In particular, a broad range of antibiotics can affect both gram-positive and gram-negative bacteria, resulting in imbalanced gut microbiota homeostasis [186]. This possibility should be fully considered in the development of AD antibiotic therapies. Unfortunately, there are no specific antibiotics for gram-negative bacteria in clinical trials for AD treatment. As the LPS from gram-negative bacteria has a remarkable adverse effect on AD, it would be important to develop a drug that not only targets gram-negative bacteria but also neutralizes the secreted/remaining endotoxin. Consequently, to minimize the side effects of existing broad-spectrum antibiotics, multispecific-target antibiotics, which target AD-specific gram-negative bacteria and their LPS, must be used.

**Table 5:** Therapeutic approaches for Alzheimer’s disease: focusing on microbiota and gram-negative bacteria-derived molecules

Therapeutic methods	Treatment or drug	Subject or model	Target (or antibiotic range)	Effects or trial phase	References
Antibiotics	Doxycycline	APP/PS1 mice	Gram-positive bacteria Gram-negative bacteria	Cognitive dysfunction↓ Neuroinflammation↓	[187]
	Gentamicin, Vancomycin, Metronidazole, Neomycin, Ampicillin, Kanamycin, Colistin, and Cefaperazone	APP/PS1 mice	Gram-positive bacteria Gram-negative bacteria	Aβ deposition↓ Soluble Aβ↓ Neuronal loss↓ Gliosis↓	[188]
	Rifampicin	AD patients	Gram-positive bacteria Gram-negative bacteria	Phase 2	NCT03856359
	Doxycycline Rifampicin	AD patients	Gram-positive bacteria Gram-negative bacteria	Phase 3	NCT00439166
	Doxycycline Rifampicin	AD patients	Gram-positive bacteria Gram-negative bacteria	Phase 3	NCT00715858
	Minocycline	AD patients	Gram-positive bacteria Gram-negative bacteria	Phase 2	NCT01463384
	Doxycycline Rifampicin	AD patients	Gram-positive bacteria Gram-negative bacteria	Dysfunctional behavior↓ Cognitive dysfunction↓	[189]
Gingipain inhibitor	COR271 COR286 COR388	BALB/c mice	Gingipain	Aβ deposition↓ TNF-α↓ Neuronal loss↓	[59]
	COR388	AD patients	Gingipain	Phase 2/3	NCT03823404
Probiotics	*Lactobacillus acidophilus* *Bifidobacterium bifidum* *Bifidobacterium* *longum*	Aβ-administered rats	Intestinal microbiota	Cognitive dysfunction↓ LTP↑	[190]
	*Bifidobacterium longum* *Lactobacillus acidophilus*	APP/PS1 mice	Intestinal microbiota	Cognitive dysfunction↓ Aβ deposition↓	[191]
	*Lactobacillus plantarum* MTCC1325	D-galactose-induced AD albino rats	Intestinal microbiota	Aβ deposition↓ NFT↓ Cognitive dysfunction↓ Acetylcholine level↑	[192]
	*Lactobacillus acidophilus, Lactobacillus casei, Bifidobacterium bifidum, Lactobacillus fermentum*	AD patients	Intestinal microbiota	MMSE score↑	[193]
	*Lactobacillus casei W56, Lactococcus lactis W19, Lactobacillus acidophilus W22, Bifidobacterium lactis W52, Lactobacillus paracasei W20, Lactobacillus plantarum W62, Bifidobacterium lactis W51, Bifidobacterium bifidum W23, Lactobacillus salivarius W24*	AD patients	Intestinal microbiota	Systemic inflammation↓	[194]
	*Lactobacillus acidophilus, Bifidobacterium bifidum, Bifidobacterium longum*	AD patients	Intestinal microbiota	MMSE score↑	[195]
	*Lactobacillus fermentum, Lactobacillus plantarum, Bifidobacterium lactis Lactobacillus acidophilus, Bifidobacterium bifidum, Bifidobacterium longum*	AD patients	Intestinal microbiota	Cognitive dysfunction-	[196]
Intestinal microbiota reconstruction	Mediterranean-style diet	MCI patients	Non-specific bacteria	Changes of the microbiota	[157]
	Mediterranean-style diet	MCI patients	Non-specific bacteria	Changes of the microbiota	[197]
	Curcumin	APP/PS1 mice	Non-specific bacteria	Cognitive impairment↓ Aβ deposition↓ Changes of the microbiota	[198]
	Folate and vitamin B-12	Aβ-administered rats	Non-specific bacteria	Changes of the microbiota	[199]
	Ginsenoside Rg1	Tree shrew model of AD	Non-specific bacteria	Aβ deposition↓ Phosphorylated tau↓ Pro-apoptotic factor↓ Changes of the microbiota	[200]
	*Streptococcus thermophilus, Bifidobacteria longum, Bifidobacteria breve, Bifidobacteria infantis*, *Lactobacillus acidophilus, Lactobacillus plantarum,Lactobacillus paracasei, Lactobacillus delbrueckii subsp, Bulgaricus, Lactobacillus brevis*	3xTg mice	Intestinal microbiota	Cognitive impairment↓ Aβ deposition↓ Neuronal loss↓	[201]
	NK46 (*Bifidobacterium longum*) oral administration	5xFAD mice	Gram-negative bacteria	Pro-inflammatory cytokines↓ LPS↓ Gliosis↓ Neuronal loss↓ Aβ↓ Cognitive dysfunction↓	[23]
	Fecal microbiota transplant	ADLP^APT^ mice	Microbiota dysbiosis	Aβ deposition↓ NFT↓ Neuroinflammation↓ Cognitive dysfunction↓	[202]
	Fecal microbiota transplant	AD patients	Microbiota dysbiosis	MMSE score↑	[203]
	Fecal microbiota transplant	AD patients	Microbiota dysbiosis	Phase 1	NCT03998423

*Ach* Acetylcholine, *AD* Alzheimer’s disease, *Aβ* Amyloid-β, *LPS* lipopolysaccharides, *LTP* long-term potentiation, *MCI* mild cognitive impairment, *MMSE* mini-mental state examination, *NFT* neurofibrillary tangles, *NF-kB* nuclear factor-κB, *SADAScog* standardized Alzheimer’s disease assessment scale cognitive subscale

### Gingipain inhibitor for AD treatment

*P. gingivalis* is a typical gram-negative bacterium that exerts a broad and powerful effect on AD pathogenesis [104]. Gingipain, one of the byproducts of *P. gingivalis*, is a novel therapeutic target for AD treatment, which is associated with AD-related pathologies, such as Aβ and tau pathology, neuroinflammation, and neurodegeneration (Table 3). Indeed, the use of selective inhibitors for gingipain can significantly reduce AD pathology [204]. For instance, COR388, a gingipain inhibitor, is currently under a phase 3 clinical trial (NCT03823404) (Table 5). Taken together, the bacterial exotoxin-specific drugs, such as gingipain inhibitors, can be an attractive therapeutic strategy, as they can simultaneously reduce and inhibit AD-related bacteria and bacterial exotoxin, respectively.

### Probiotics for AD treatment

Probiotics have beneficial effects including immune system modulation, synthesis and release of neurotransmitters, protection from physiological stress, host gene expression modulation, pathogen antagonism, and improvement of intestinal epithelial barrier function [205]. Moreover, the hippocampal expression of N-methyl-D-aspartic acid receptor, which is very important in AD pathology, is regulated by gut microbiota [206]. Several studies have suggested the potential therapeutic effect of probiotics in AD (Table 5). Surprisingly, many studies demonstrated that the probiotic treatment in rodent models of AD can reduce Aβ plaques and NFT [191, 192], alleviate neurodegeneration [190, 207], and restore the reduced acetylcholine level [192]. Furthermore, probiotics restore cognitive dysfunction in AD rodent models [190–192]. Evidence for the improvement of AD-related pathology by probiotics has been reported both in AD animal models and in patients. A clinical trial conducted in patients with AD has reported that a 12-week probiotic administration significantly improves the cognitive function in AD patients [193, 195]. The probiotic administration to AD patients has also been reported to alleviate systemic inflammation by reducing intestinal inflammation [194]. The effects of probiotics both in AD animal models and patients might occur through direct probiotic bacterial interaction with AD pathology and the correction of AD-induced microbial dysbiosis. Disruption of microbiota homeostasis, which is maintained through competition between bacterial species, could lead to pathological conditions. Attempts to rebuild the gut microbiota through dietary modulation and intake of food components are receiving attention in the treatment of AD. Modulating the microbiota bias is an important factor in the treatment of many diseases. Considering the changes in gram-negative bacteria in AD (Table 1), the mechanisms of action of probiotics on AD may also include a probiotic antagonistic action against the dysbiosis of gram-negative bacteria.

### Intestinal microbiota reconstruction for AD treatment

Microbiota dysbiosis is an important factor in AD-related pathogenesis and progression [208], and several attempts have been made to improve microbiota dysbiosis and the alteration of gram-negative bacteria in AD (Table 5). First, attempts to induce the rebuilding of the gut microbiota through intake of the diet and food components are receiving attention for the treatment of AD [209]. For instance, a Mediterranean-style diet, which emphasizes plant-based foods such as vegetables, beans, whole grain, fruits, nuts and seeds, and plant-based oils [210], was reported to modulate the gut microbiota affecting AD pathology [197, 211]. One study demonstrated that the gut microbiota distribution alters in MCI patients on a Mediterranean diet, particularly decreasing the abundance of gram-negative bacteria *Enterobacteriaceae*, *Akkermansia*, *Christensenllaceae*, and *Erysipelotriaceae* [211]. Moreover, curcumin can improve AD pathology by regulating the proportion of gram-negative bacteria such as *Bacteroidaceae*, *Rikenellaceae*, and *Prevotellaceae* in AD transgenic mice [198]. Similarly, supplementation of omega-3 fatty acid and DHA alleviates microbiota dysbiosis and reduces AD-related gram-negative bacteria, such as *Bacteroidetes,* in healthy individuals [212, 213]. Vitamins are closely correlated with microbiota, and intake of folate and vitamin B-12 has been reported to be important for intestinal microbiota homeostasis in a rodent AD model [199, 209]. Moreover, traditional herbal medicine can induce changes in the microbiota in AD. The Ginsenoside Rg1, a traditional herbal medicine, can affect the microbiota of the large intestine by significantly reducing the abundance of gram-negative bacteria, *Bacteroidetes*, in the tree shrew model of AD [200]. Second, microbiota or fecal transplantation, which involves transplantation of microbiota in the feces of healthy humans into patients to balance the intestinal microflora, is an emerging therapeutic method for AD treatment [203]. Microbiota modulation both reduces cognitive impairment and Aβ aggregates, and restores the impaired neuronal proteolytic pathways in 3xTg mice [214]. Moreover, recent studies have reported therapeutic effects of microbiota transplantation, including reduction of Aβ deposition and NFT, alleviation of neuroinflammation, and amelioration of cognitive decline in ADLP^APT^ mice [215]; and alleviation of behavioral and psychological symptoms of dementia and continuous improvement of cognitive function in elderly patients with AD who received fecal transplants [216]. However, the safety of fecal transplantation remains controversial. Recently, a patient who underwent fecal transplantation died from *E. coli* infection, a gram-negative bacterium that secrete “extended-spectrum beta-lactamase” [217]. This suggests that the transplantation of microbiota—including gram-negative bacteria—is an unstable AD treatment. Despite the controversy on stability and side effects, the reconstruction of microbiota distribution through fecal transplantation has relieved the AD-related pathology in both animals and patients with AD. These results suggest that the microbiota, including gram-negative bacteria, may not only be an upstream etiology of AD onset and progression, but also a therapeutic target for AD treatment.

## Conclusions

The gram-negative bacteria and their LPS are detected in the CNS as well as in the periphery, and can trigger or accelerate AD pathology. We discuss the alterations and species of gram-negative bacteria in AD (Tables 1 and 2). The gram-negative bacteria can directly penetrate the CNS through various mechanisms (Fig. 1) and influence AD pathogenesis (Fig. 2). Moreover, several gram-negative bacteria are involved in microbiota dysbiosis, Aβ pathology, tau hyperphosphorylation, neuroinflammation, and neurodegeneration in AD. Furthermore, the impact of gram-negative bacterial byproducts on major AD pathologies suggests that the gram-negative bacteria are an essential therapeutic target for AD (Table 3). Importantly, gram-negative bacteria-derived LPS, which is present at high concentrations in AD patients, is a direct pathogenic factor (Fig. 4). The AD pathology-related localization of LPS within the CNS suggests that LPS has unique pathological roles in AD (Table 4). Moreover, LPS is directly involved in AD pathology, including neuroinflammation through microglial TLR4 and induction of neuronal cell death through neuronal TLR4. The ‘LPS cascade phenomenon’, which acts as an upstream molecule triggering AD pathogenesis or accelerating progression by engagement in various aspects of AD pathology, should be considered as a potential therapeutic target for AD treatment. As a novel therapeutic strategy for AD, the modulation of LPS-releasing gram-negative bacteria is receiving much attention (Table 5). Although the bacteria-targeting treatments, such as antibiotics and fecal microbiota transplantation, show potential for AD treatment, there are still concerns regarding their side effects and safety. In particular, potential side effects of the use of non-specific drugs that target bacteria indiscriminately should receive cautions. Therefore, it is important to categorize and characterize gram-negative bacteria that affect AD. Taken together, the gram-negative bacteria and their LPS are not only an upstream pathologic process which influences Aβ and tau pathology, but are also attractive targets for AD treatment. With no practical treatment for AD yet in development, the control of gram-negative bacteria and their LPS may be an excellent strategy to prevent the onset and progression of AD.

## Supplementary Information

**Additional file 1: Figure S1**. Flow diagram showing the study selection process. Flowchart summarizing study selection and inclusion processes in this narrative review, including the example of keywords that were reviewed.

## Data Availability

Not applicable.

## Abbreviations

AD: Alzheimer’s disease
ADAM10: A disintegrin and metalloproteinase domain-containing protein 10
Akt: Protein kinase B
ApoE: Apolipotein E
apoER2: Apolipoprotein E receptor 2
APP/PS1: Amyloid precursor protein/presenilin 1
Aβ: Amyloid beta
BACE-1: Beta-site amyloid precursor protein cleaving enzyme 1
BBB: Blood–brain barrier
*C. pneumoniae*: *Chlamydia pneumoniae*
CD14: Cluster of differentiation 14
CDK-5: Cyclin-dependent kinase 5
CNS: Central nervous system
CSF: Cerebrospinal fluid
*E. coli*: *Escherichia coli*
Ecgp96: Endothelial receptors beta-form of the heat-shock gp96
GSk-3β: Glycogen synthase kinase-3β
*H. pylori*: *Helicobacter pylori*
IbeA: Invasion of the brain endothelium protein A
IL: Interleukin
iNOS: Inducible nitric oxide synthase
LBP: Lipopolysaccharide binding protein
LPS: Lipopolysaccharide
LRP-1: Lipoprotein receptor-related protein 1
MAPK: Mitogen-activated protein kinase
MG: Methylglyoxal
MyD88/TRIF: Myeloid differentiation primary response 88/toll/interleukin-1 receptor-domain-containing adapter-inducing interferon-β
NFT: Neurofibrillary tangles
NF-κB: Nuclear factor kappa-light-chain-enhancer of activated B
OmPA: Outer membrane protein A
OMV: Outer membrane vesicle
*P. gingivalis*: *Porphyromonas gingivalis*
PI3K: Phosphoinositide-3-kinase
RAGE: Receptor for advanced glycation end products
SCFA: Short-chain fatty acids
TLR: Toll-like receptor
TREM2: Triggering receptor expressed on myeloid cells 2
VacA: Vacuolating cytotoxin A
