# Supplementary figures and images for: Gram-negative bacteria and their lipopolysaccharides in Alzheimer’s disease: pathologic roles and therapeutic implications
Source: Transl Neurodegener. 2021 Dec 7;10:49. doi: 10.1186/s40035-021-00273-y (PMC8650380; doi:10.1186/s40035-021-00273-y)

## Slide 1
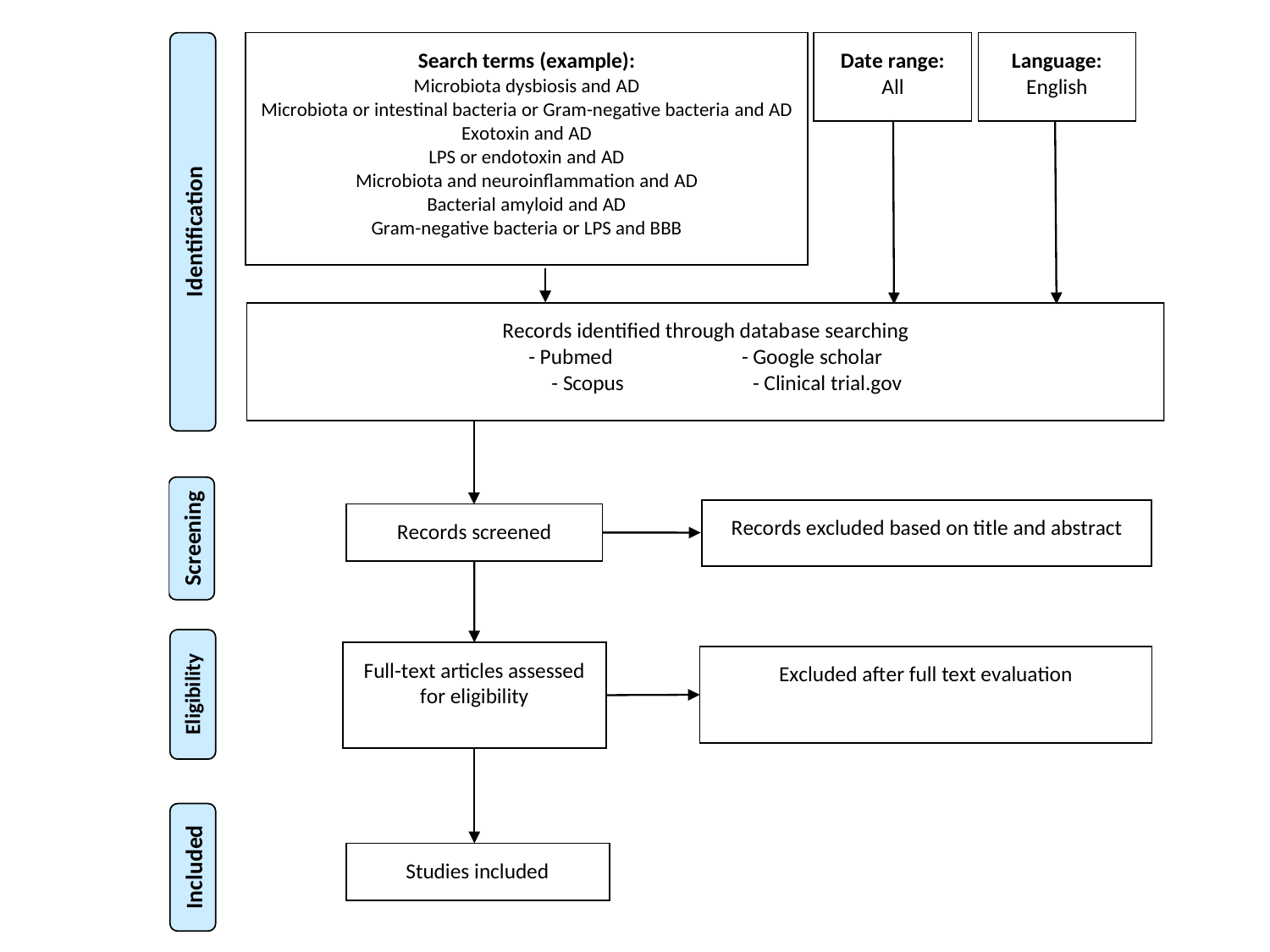

Supplement: Supplementary file 1 — Additional file 1: Figure S1. Flow diagram showing the study selection process. Flowchart summarizing study selection and inclusion processes in this narrative review, including the example of keywords that were reviewed. [file 40035_2021_273_MOESM1_ESM.pptx]
